# Clinical application of 90-gene expression test in a patient with occult breast cancer: a case report and literature review

**DOI:** 10.1007/s12282-025-01728-0

**Published:** 2025-06-05

**Authors:** Guojie Xu, Kewei Zhao, Xi Zhou, Liling Zhang, Jiaying Liu, Yanxia Zhao, Dan Han

**Affiliations:** Cancer Center, Union Hospital, Tongji Medical College, Huazhong University of Science and Technology, Wuhan, China

**Keywords:** Occult breast cancer, Diagnosis, Genetic testing, 90-gene expression test, Endocrine therapy

## Abstract

Occult breast cancer is an exceptionally rare disease characterized by ambiguous diagnostic criteria. Currently employed diagnostic methods include breast MRI, PET-CT, and immunohistochemistry; however, challenges in achieving accurate diagnoses persist in certain cases. The utilization of the 90-gene test has been implemented to identify the origin of unknown tumors, demonstrating an overall accuracy rate of 94.4% and sensitivity ranging from 74.2% to 100%, as evidenced by clinical trials. In this particular case, the patient is presented with both occult breast cancer and thyroid cancer. Initial imaging results indicated thyroid cancer without evident breast nodules or axillary lymph node involvement. Despite treatment for thyroid cancer, the patient’s pain did not alleviate until tissue specimens were subjected to a comprehensive analysis using the 90-gene expression test. The gene expression profiling revealed a similarity score of 59.5%, confirming the diagnosis of occult breast cancer as a Luminal B subtype. Following treatment with letrozole and norethindrone, significant relief was observed in the patient’s pain, along with a notable reduction in the size of liver metastases and mediastinal lymph node lesions. This case highlights the immense potential of the 90-gene test as a diagnostic tool for occult breast cancer.

**Supplementary Information:**

The online version contains supplementary material available at 10.1007/s12282-025-01728-0.

## Introduction

In 2022, the incidence of breast cancer in China reached 511,700 cases, ranking second only to lung cancer, which had 581,800 cases, thereby making breast cancer the second most prevalent cancer among Chinese women [1]. With advancements in screening methods and the emergence of various treatment options such as novel endocrine therapy, targeted therapy, or antibody–drug conjugates (ADC) therapeutic drugs, the mortality rate from breast cancer was reported at 108,600 deaths among patients, placing it fifth among female cancer-related mortalities [1]. Among them, occult breast cancer (OBC) is a type of breast cancer that is relatively difficult to diagnose. It commonly presents with initial symptoms and primary manifestations of lymph node metastasis in the axilla or other distant sites, often without detectable primary tumors through physical examination or imaging tests, leading to a higher risk of missed diagnosis or misdiagnosis [2, 3]. Given its low incidence rate, many oncologists lack sufficient knowledge regarding OBC. Magnetic resonance imaging (MRI) is a crucial diagnostic tool for OBC due to its high-resolution capabilities, which enable the detection of minute tumor lesions that are unidentifiable through alternative approaches, while maintaining high sensitivity and specificity for breast cancer detection. In addition, 18F-fluorodeoxyglucose positron emission tomography/computed tomography (18F-FDG-PET/CT) can be employed when necessary [4–6]. Given the low incidence rate of OBC, many oncologists lack sufficient knowledge regarding this condition. Immunohistochemical detection plays an instrumental role in determining the origin of primary tumors. Breast cancer tissue specimens typically exhibit positive results for CK7, GATA-3, GCDFP15, and SCGB2A2, with approximately 30% of OBCs testing positive for estrogen receptor (ER) and progesterone receptor (PR), while 40% are negative [7, 8].

This paper presents a unique case of concurrent thyroid cancer and occult breast cancer, which remained undiagnosed despite extensive evaluation using breast ultrasound, breast MRI, ^18^F-FDG-PET/CT, and immunohistochemistry. The identification of occult breast cancer was ultimately achieved through tumor origin gene detection. This is the first reported case utilizing 90-gene expression technology for diagnosing occult breast cancer, providing valuable insights for its diagnosis.

## Case presentation

**Fig. 1:**
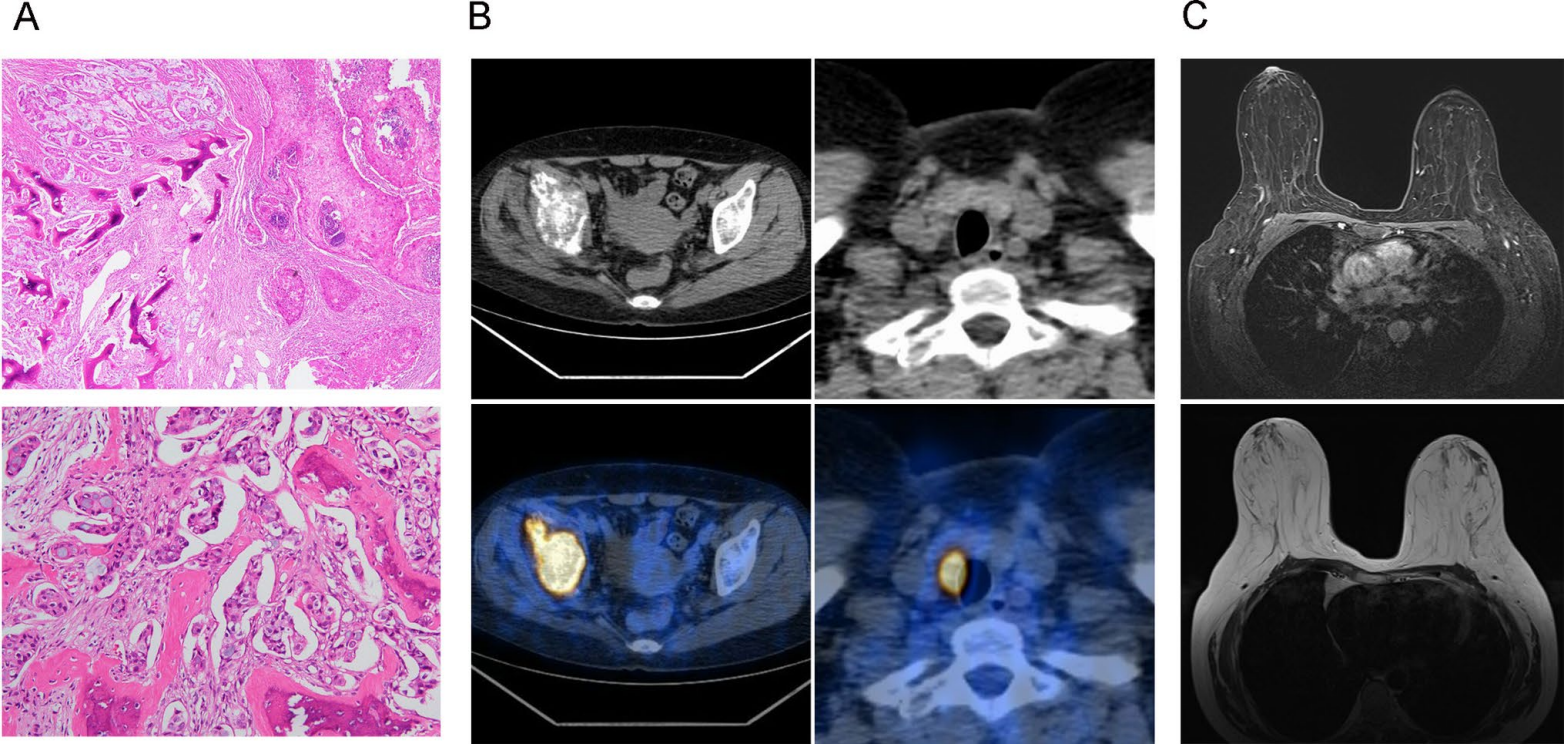
Baseline imaging and pathologic results. **A** Hematoxylin and eosin staining on right ilium lesion under puncture biopsy showed metastatic adenocarcinoma. **B** High uptake of fluorodeoxyglucose (FDG) in right ilium and right thyroid lobe. **C** MRI examination of the breast showed no obvious tumor lesions

A 56-year-old Chinese woman presented with unprovoked right hip joint pain in August 2022, without any specific intervention. In January 2023, the patient reported an exacerbation of the pain. On February 12, 2023, a pelvic MRI was performed at the Orthopedics Department of Huazhong University of Science and Technology Affiliated Union Hospital, revealing: (1) significant osseous destruction in the right ilium suggestive of metastatic malignancy; (2) reduced density in the neck of the right femoral head indicating potential tumor involvement. Subsequently, on February 16, 2023, a biopsy of the right ilium was conducted in the Bone Tumor Department of our hospital, which confirmed metastatic adenocarcinoma upon postoperative pathological examination (Fig. 1A). The immunohistochemical analysis demonstrated positive expressions for PCK, CK7, and ER, partial positivity for CK20 and P16, and negative expressions for SOX10, WT-1, PAX8, TTF-1, CDX2, and Villin. Those immunohistochemical staining suggested a likely breast origin and recommended further comprehensive clinical investigations for precise diagnostic clarification. On March 3, ultrasound examinations were performed to evaluate both breasts and the thyroid gland, revealing no abnormalities in the bilateral breasts but identifying a solid nodule categorized as ACR TI-RADS category 5 within the right lobe of the thyroid gland, along with a cystic-solid nodule categorized as ACR TI-RADS category 3 within the left lobe. 18F-FDG PET/CT findings indicate the following: (1) destruction of bone tissue and metabolic abnormalities in the right ilium and ischium, with increased metabolism suggestive of a metastatic lesion; (2) no significant focal hypermetabolism observed in the breast region bilaterally; (3) a metabolically hyperactive nodule in the lower part of the right thyroid lobe, suggesting a potential malignant tumor (Fig. 1B). MRI of the breast reveals: (1) a high T2 signal nodule in the lateral lower part of the left breast, suggestive of a small fibroadenoma with possible mucinous changes; (2) no abnormal axillary lymph nodes identified (Fig. 1C).

A multidisciplinary team (MDT) comprising the departments of oncology, endocrine surgery, medical oncology, and radiology discussed the patient’s condition on March 20, 2023. The MDT’s recommendations are as follows: (1) the current diagnosis is considered thyroid cancer; (2) surgical intervention is recommended. The patient underwent a “total thyroidectomy (bilateral) + autologous parathyroid gland transplantation (left)” on March 23, 2023. Postoperative pathology reveals: (1) multiple focal micropapillary carcinoma of the thyroid (two lesions, sizes: 1.1 cm × 0.8 cm and 0.2 cm in diameter, histological subtype: classical type); (2) microscopic papillary carcinoma of the thyroid (single lesion, diameter: 0.2 cm, histological subtype: classical type); (3) LYMPH node metastasis observed in the left central group (1/4) and right central group (2/3) with the maximum metastasis size being 0.2 cm, and extra-capsular invasion noted (Supplementary Fig. 1A and 1B). The patient continued postoperative treatment with levothyroxine and calcium tablets.

**Fig. 2:**
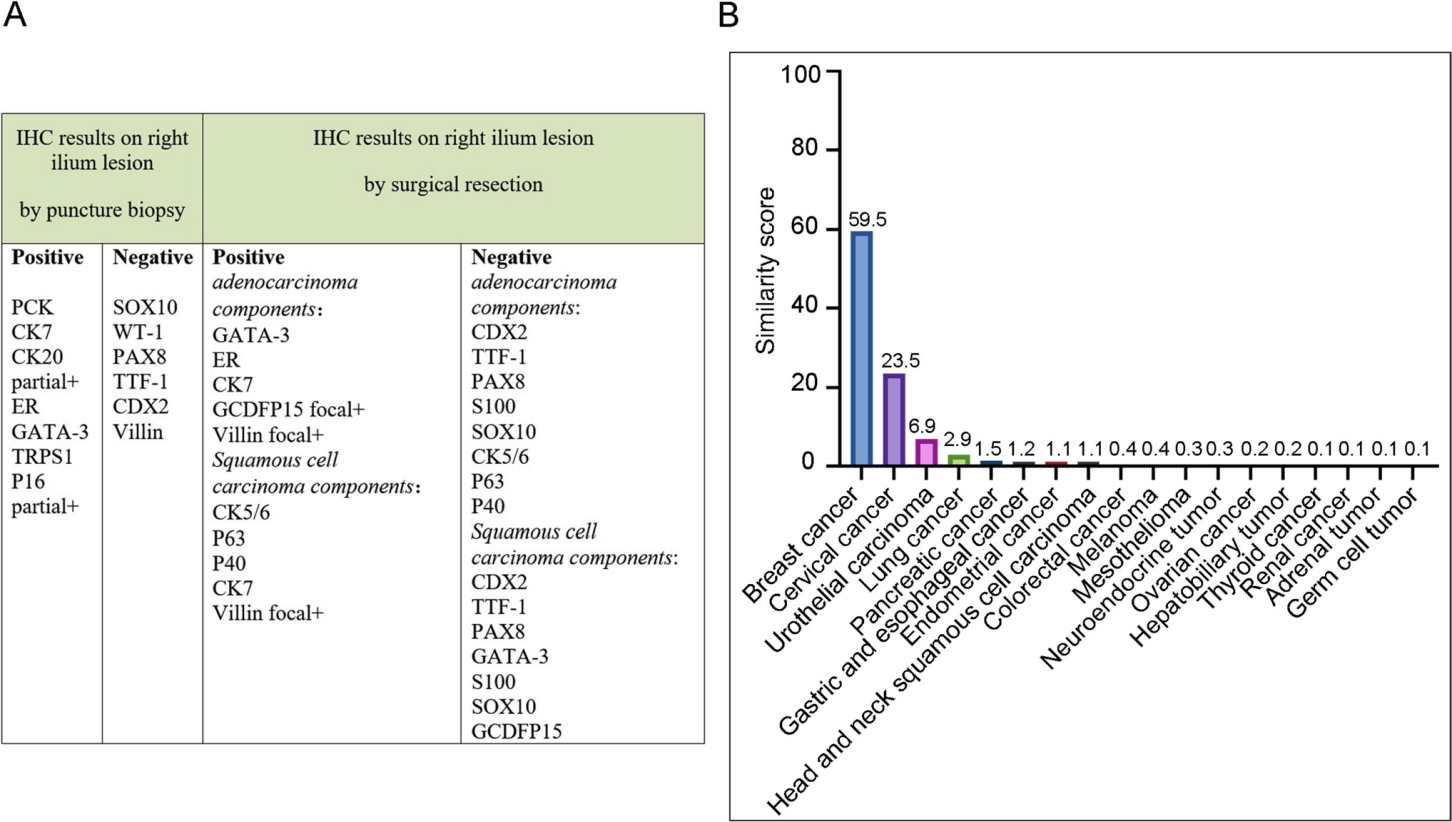
Summary of pathological and molecular pathological information. **A** Immunohistochemical (IHC) results of biopsy tissue and surgical excision tissue. **B** 90-gene tumor tissue traceability expression assay results. The maximum similarity score for tumor tissue traceability was 100

In May 2023, the patient presented progressive bilateral pelvic pain and immobility. On May 10, 2023, she underwent a surgical procedure for “resection and reconstruction of pelvic tumor along with total hip joint replacement” at the orthopedic department. The postoperative histopathological examination revealed invasive/metastatic adenosquamous carcinoma. The immunophenotype did not indicate any specific organ involvement. Immunohistochemistry staining demonstrated positive expression of GATA-3, ER, CK7, and focal positivity for GCDFP15 and Villin; negative expression was observed for CDX2, TTF-1, PAX8, S100, SOX10, CK5/6, P63, and P40 in adenocarcinoma components. Squamous cell carcinoma components exhibited positive staining for CK5/6, P63, P40, and CK7; focal positivity for Villin; negative expression of CDX2, TTF-1, PAX8, GATA-3, S100, and SOX10; and absence of GCDFP15 (Fig. 2A). Subsequently, the patient developed complications including impaired wound healing and infection, resulting in a subsequent “debridement followed by suture coverage using artificial skin” procedure on August 16th, 2023. After this intervention, the patient received anti-inflammatory treatment along with supportive care. In December 2023, the patient reported experiencing back pain. Following an enhanced CT scan conducted at our hospital’s oncology department on December 19th,2023, the following findings were noted: (1) multiple mediastinal lymph nodes displayed enlargement in size; (2) several liver nodules were considered as potential metastatic tumors; (3) pelvic reconstruction was performed along with a right hip replacement due to multiple rib and vertebral bone destruction, including a pathological fracture in the Th5 vertebra, which was indicative of metastatic bone disease. Enhanced computed tomography (CT) scans revealed the presence of new lesions in the liver and mediastinal lymph nodes.

**Fig. 3:**
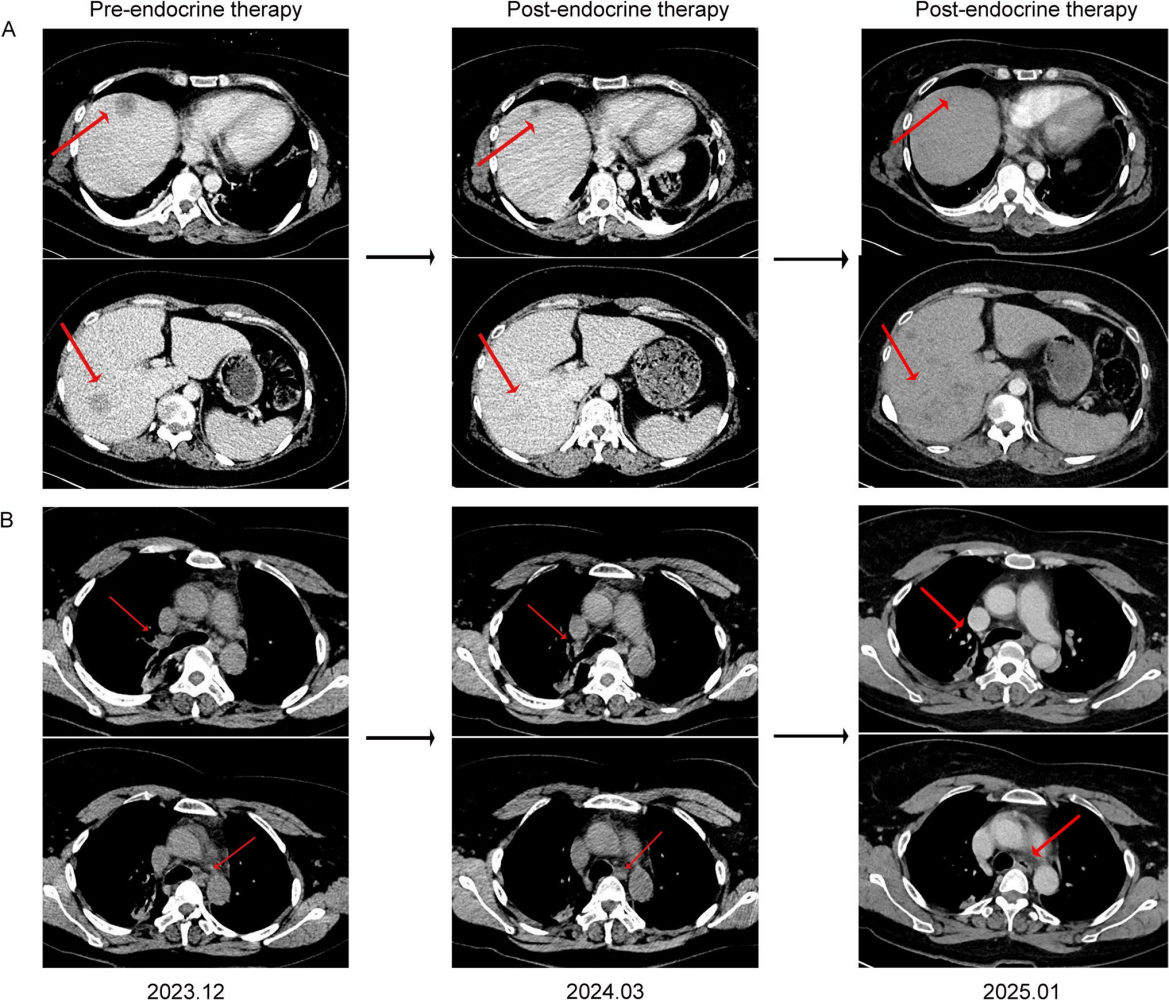
**A** Computed tomography scans of metastatic hepatic nodules at baseline (left) and after endocrine therapy (right). **B** Computed tomography scans of metastatic mediastinal lymph nodes at baseline (left) and after endocrine therapy (right)

**Fig. 4:**
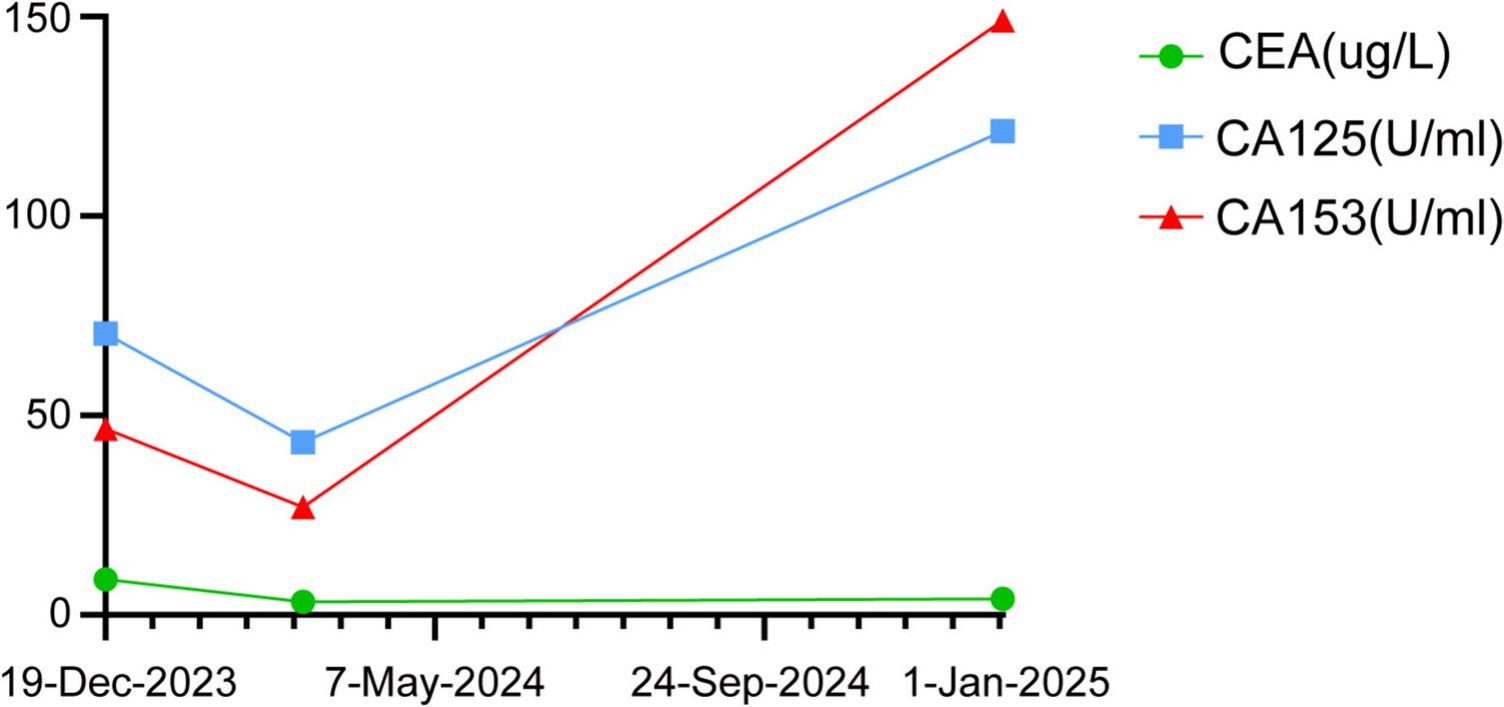
The curve of tumor markers of CEA, CA125, CA153 over time, from December 21, 2023, to January 1, 2025

As the primary tumor could not be identified, a tumor tissue origin gene test was conducted on December 21, 2023, using surgical specimens collected from pelvic tumor of the patient for tumor cell isolation. Subsequently, RNA extraction from these cells was performed, followed by reverse transcription and polymerase chain reaction (PCR) amplification. The gene detection results demonstrated elevated levels of breast cancer-related genes, including AZGP1, GATA-3, KRT14, XIST, and ESR1. Gene expression profiling confirmed that the primary tumor originated from breast cancer (Fig. 2B and Supplementary Table 1). To further validate this finding, hematoxylin and eosin (HE) staining was repeated by the pathology department, alongside immunohistochemistry analysis, which revealed ER positivity (60%, strong–moderate), PR positivity (30%, strong-moderate), HER2 positivity (2 + , FISH negative), and a Ki67 labeling index of 20%. Consequently, our team initiated treating this patient with abemaciclib in combination with letrozole and norethindrone. The patient experienced a significant reduction in pain after 1 week of treatment. An enhanced CT scan on March 12, 2024, showed that the sizes of the mediastinal lymph nodes and liver metastatic lesions were slightly reduced. On January 1, 2025, an enhanced CT scan indicated those metastatic lesions were stable (Fig. 3). The tumor makers of CEA, CA125, and CA153 significantly decreased on March 12, 2024 and increased on January 1, 2025 (Fig. 4). The patient is currently undergoing further treatment.

## Discussion

Currently, there are no definitive diagnostic criteria for occult breast cancer. The American Radiological Association recommends breast MRI as the preferred diagnostic modality for this condition [9]. Breast MRI exhibits superior sensitivity (over 72%) compared to conventional imaging techniques such as CT and ultrasound [10, 11]. Furthermore, 3.0 T breast MRIs provide significant advantages over 1–1.5T breast MRIs in terms of tissue resolution and noise reduction [12]. In addition, ^18^F-FDG PET/CT serves as a whole-body imaging approach that assists in identifying the primary tumor; however, its diagnostic value is limited for tumors with low FDG uptake or small lesions due to reduced sugar metabolism within the tumor [12]. Notably, despite negative findings on ultrasound, MRI, and PET-CT scans regarding abnormal lesions in the breasts or axillary lymph nodes, an incidental malignant thyroid tumor was detected which further complicated early tumor diagnosis.

Breast cancer heterogeneity significantly influences the diagnosis and treatment of the disease. The established molecular subtypes of breast cancer—luminal A, luminal B, HER2 positive, and triple-negative breast cancer (TNBC)—have been well characterized. Advances in RNA sequencing (RNA-seq) technology now allow for the analysis of various genetic aspects of breast cancer cells, elucidating the complex interactions between these cells and their microenvironment. This progress facilitates a more comprehensive classification of breast cancer at the genetic level [13]. While metastatic tumors exhibit gene expression profiles that are distinct from those of their tissue of origin, they also share similarities with the gene expression patterns observed in the tissue of the metastatic site. These findings indicate that tumors retain certain molecular characteristics associated with their tissue of origin throughout their development, progression, and metastasis [14]. By detecting 90 characteristic genes using gene chip technology or RNA sequencing analysis, it is feasible to determine both the tissue origin and specific type of cancer present in a given sample by assessing the expression levels of these genes. This detection method has demonstrated an overall diagnostic agreement rate of 90% for distinguishing among 21 common tumor types [8, 15]. In a large multicenter clinical study initiated by Sun Wei’s team at Cancer Hospital of Chinese Academy Medical Sciences involving 1471 patients across 21 different types of cancers, screening for these 90 characteristic genes achieved an impressive overall accuracy rate of 94.4%, with sensitivities ranging from 74.2% to 100%. Notably, high sensitivities were observed for lung cancer (95%), breast cancer (98.4%), colon cancer (93.9%), and gastric cancer (90.6%), respectively [16]. Recently, Wang et al. conducted gene screening on 90 genes in a cohort of 112 patients diagnosed with TNBC. The assay accurately identified the primary site in 97.6% of cases (41 out of 42) with lymph node metastases and distant metastatic tumors were correctly detected in 96.8% of cases (30 out of 31). Subsequently, they identified TNBC-specific genes including AZGP1, KRT19, and PIGR [17].

The isolation of tumor cells is the most critical step in determining the 90-gene tests. The isolation from formalin-fixed, paraffin-embedded (FFPE) tissues presents unique opportunities and significant challenges in translational cancer research. Although FFPE archives are an invaluable resource for retrospective studies, the technical limitations imposed by fixation-induced biomolecular damage require careful methodological optimization and interpretation of results. A primary concern in FFPE-based studies is the cross-linking and fragmentation of nucleic acids caused by prolonged formaldehyde fixation. While some researchers argue that these artifacts preclude reliable high-throughput sequencing [24]. They demonstrate that prolonged protease K digestion combined with heat-induced antigen retrieval (HIAR) can partially reverse these effects, thereby enabling robust recovery of DNA and RNA. The choice of tumor cell isolation is between enzymatic digestion and mechanical dissociation, influenced by the composition of the tissue analyzed. Enzymatic methods tend to produce higher cellularity in fibrotic tumors, such as pancreatic adenocarcinoma; however, these methods are associated with an increased risk of DNA shearing. In contrast, mechanical dissociation techniques are more effective in preserving high-molecular-weight DNA, yet they are limited by lower throughput. Our tissues used for gene test has a higher number of tumor cells, making the mechanical method preferable.

In this case, the patient was diagnosed with two malignant tumors: occult breast cancer and thyroid cancer. Bone biopsy specimens suggested the presence of breast cancer, while imaging results indicated thyroid cancer. Despite treatment for thyroid cancer, the patient did not achieve pain relief. Consequently, the patient’s tissue specimens underwent expression detection for 90-gene signatures. The results revealed elevated levels of several breast cancer-related genes, including AZGP1, GATA-3, KRT14, XIST, and ESR1. The gene detection results yielded a similarity score of 59.5% for breast cancer. In addition, the tissue specimens were assessed for ER, PR, HER2, and Ki67, leading to the classification of the patient as luminal B type.

Matthew H. Larson and his team identified tumor-specific transcripts by utilizing RNA background-based gene expression from healthy blood cells. Larson coined the term “dark channel biomarkers (DCB)” to refer to gene regions exhibiting no background expression in circulating blood cells. DCB genes are significantly enriched in tissue-specific genes and correlate with the observed cancer types; for instance, breast-specific DCBs were detected in the plasma of breast cancer patients. This finding suggests that RNA may serve as a predictor of tumor tissue origin. In certain patient samples, low-grade tumors exhibited high tissue expression of SCGB2A2 in plasma, while others demonstrated elevated expression levels of both SCGB2A2 and FABP7 [18]. However, SCGB2A2 sensitivity is limited particularly for detecting triple-negative breast cancer; moreover, it has been reported to be present in female reproductive tract tumors such as cervix, endometrium, ovary as well as certain salivary gland tumors and skin cancer. Combining SCGB2A2 with GATA-3 and GCDFP15 can enhance detection accuracy especially when determining if metastatic adenocarcinoma originates from the breast [19, 20]. Helene et al. found that ANPEP, AZGP1, and SPDEF were three of the genes identified as overexpressed in breast cancer compared to ovarian cancer [21]. The expression of AZGP1 in breast cancer tissues was negatively correlated with the survival of breast cancer patients [22]. The rapid emergence of liver and mediastinal lymph node metastases during treatment suggests the presence of adverse prognostic genes, which also carry prognostic implications. However, gene sequencing has some drawbacks such as complex operations, high requirements for DNA concentration and sample purity, unstable samples, and vast amounts of data that limit its clinical application. It is believed that technological advancements will lead to better development in the future.

The treatment principles for occult breast cancer (OBC) are fundamentally aligned with those for invasive breast cancer. A comprehensive treatment plan should be tailored to the individual, emphasizing local interventions such as surgery and radiation therapy, while also recognizing the significance of systemic therapies, including chemotherapy, endocrine therapy, targeted therapy, and immunotherapy [23]. Surgery is a cornerstone in managing occult breast cancer, involving procedures such as axillary lymph node dissection or mastectomy. Radiation therapy plays a vital role in the overall management of occult breast cancer, particularly for patients who encounter surgical challenges or wish to preserve breast tissue. For estrogen receptor-positive (ER/PR +) tumors, endocrine therapy is recommended, as the efficacy of estrogen modulators and aromatase inhibitors has been substantiated in numerous clinical trials [4]. In this case, the patient was diagnosed with ER/PR + occult breast cancer with metastasis to multiple sites and subsequently received treatment with letrozole, abemaciclib, and norethindrone. Following this treatment, the patient experienced significant pain relief and notable reductions in metastatic lesions in the liver and mediastinal lymph nodes. These findings indicate that a combination of an aromatase inhibitor and a CDK4/6 inhibitor may also be appropriate for patients with late-stage hormone-dependent occult breast cancer.

## Conclusions

Occult breast cancer is an exceptionally rare disease with ambiguous diagnostic criteria. In this particular case, the patient’s initial imaging findings showed only thyroid cancer, with no evident breast nodules or axillary lymph node involvement. Despite treatment for thyroid cancer, the patient’s pain did not alleviate until tissue specimens were subjected to a comprehensive analysis using the 90-gene expression test. The gene expression profiling revealed a similarity score of 59.5%, confirming the diagnosis of occult breast cancer as Luminal B subtype. Following treatment with letrozole and abemaciclib, significant relief was observed in the patient’s pain along with notable reduction in liver metastases and mediastinal lymph node lesions size. This case highlights that the application of the 90-gene test holds immense potential as a diagnostic tool for occult breast cancer.

## Methods

### RNA extraction

The tumor tissues used for the origin gene test came from the pelvic tumor which were collected in the surgery of “resection and reconstruction of pelvic tumor along with total hip joint replacement” on May 10, 2023. Tissue embedding was conducted in the Pathology Department of our hospital. For cases meeting the inclusion and exclusion criteria, 5 to 15 unstained sections, each 5 μm thick, were freshly cut for total tumor cell RNA isolation. Tumor cells were manually enriched through macro-dissection. Total RNA was isolated using the FFPE Total RNA Isolation Kit (Canhelp Genomics Co., Ltd., Hangzhou, China), as previously described [16]. The concentration and purity of the total RNA were measured using a spectrophotometer. The exclusion criteria included insufficient RNA, defined as total RNA concentrations of 2.1 or < 1.7.

### Gene expression profiling and classification algorithm

The 90-gene expression assay was conducted by Canhelp Genomics Co., Ltd, which was performed as previously described. In summary, reverse transcription was executed on the isolated total RNA. Following this, RT-PCR was conducted using a 7500 Real-Time PCR System (Applied Biosystems) to profile tumor-specific gene expression. The internal control (IC) gene was utilized to assess sample quality, with samples exhibiting a weak RT-PCR signal (cycle threshold [Ct] value of the IC greater than 38) being excluded from analysis. In addition, a no template control (NTC) was employed to evaluate potential contamination in the PCR reaction, and samples were excluded if the Ct of the NTC was less than 38. For each case, the 90-gene classifier analyzed the expression patterns of the 90 tumor-specific genes and generated similarity scores for each primary tumor type based on the degree of similarity between the test specimen and the gene expression database. The similarity scores ranged from 0 (indicating low similarity) to 100 (indicating high similarity) for each tumor type, with the total sum of similarity scores across 21 tumor types equaling 100.

### Statistical analysis

Statistical analysis was performed to evaluate the performance of the 90-gene expression assay. All internal accession numbers were de-identified, and test results were compared with reference diagnoses. For each tumor type included in the panel, sensitivity (or positive percent agreement) was defined as the ratio of true positive results to the total number of positive samples analyzed. Specificity (or negative percent agreement) was defined as the ratio of true negative results to the total number of negative samples analyzed. A confusion matrix was generated for each tumor type to summarize the results. All statistical analyses were conducted using R software (version 3.6.1) and GraphPad Prism 8.01. Statistical tests were two-sided, and p values less than 0.05 were deemed statistically significant.

## Supplementary Information

Below is the link to the electronic supplementary material.

Supplementary file1 (PDF 1846 KB)

Supplementary file2 (TIF 4540 KB) Supplementary Figure 1. The HE-stains results of thyroid surgery and diagnosis of papillary carcinoma of the thyroid. (A) HE*40; (B) HE*100

Supplementary file3 (XLSX 13 KB)

## Data Availability

The data are available from the corresponding author (tjxhhandan@163.com ,sophia7781@126.com) upon reasonable request. Additional data are available as supplementary material.
