# Supplementary material for: Clinical application of 90-gene expression test in a patient with occult breast cancer: a case report and literature review
Source: Breast Cancer. 2025 Jun 5;32(5):1144–51. doi: 10.1007/s12282-025-01728-0 (PMC12394298; doi:10.1007/s12282-025-01728-0)
Supplement: Supplementary file 1 — Supplementary file1 (PDF 1846 KB) [file 12282_2025_1728_MOESM1_ESM.pdf]

可帮医学
Canhelp Medical

让医生有力量 让患者有希望

肿瘤组织起源检测报告

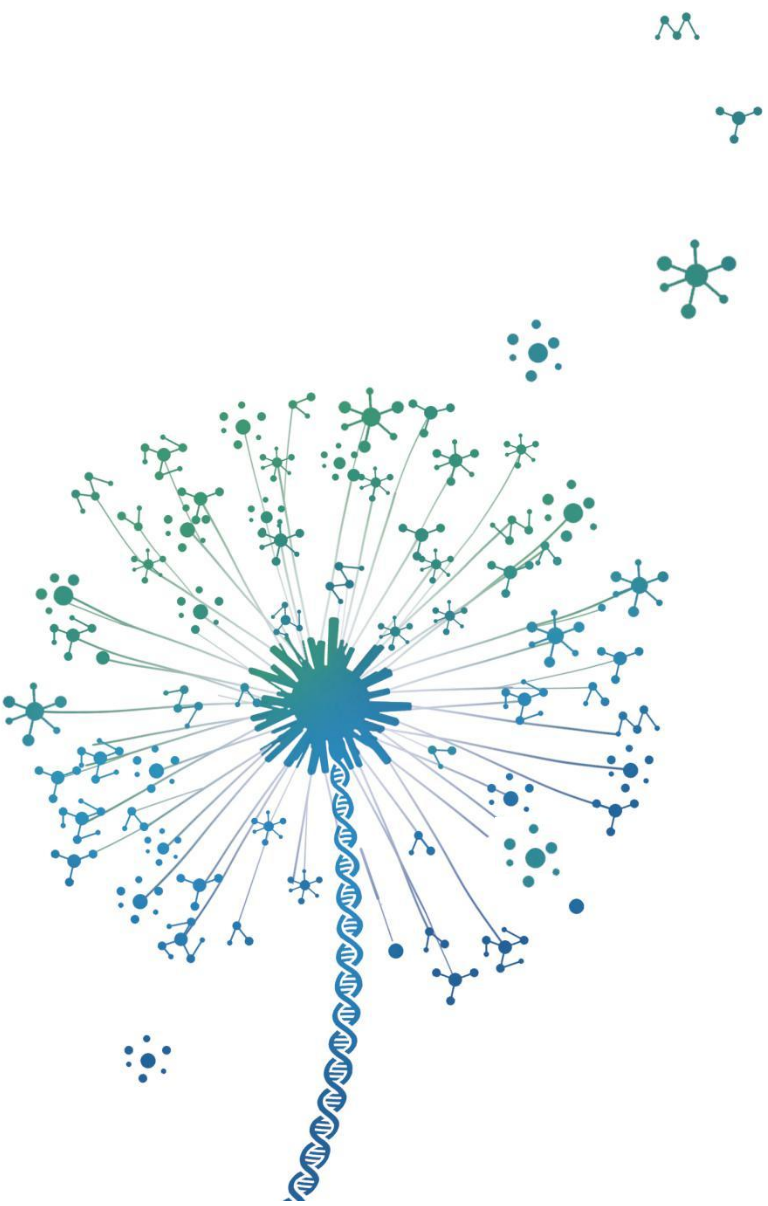

感谢您对可帮的信任，选择 Canhelp®−Origin 肿瘤组织起源检测项目

原发灶不明恶性肿瘤(Cancer of Unknown Primary，CUP)是一类经病理学诊断确诊为转移性恶性，但是通过详细评估未能明确原发位点的异质性肿瘤。据统计，CUP 约占全部肿瘤病例的 5%~10%，居常见恶性肿瘤的第 8 位，CUP 患者的预后很大程度上取决于原发肿瘤的生物学特性，因此找出肿瘤的组织起源，采取有针对性的治疗，对于改善患者预后具有重要意义。

近年来，随着生物技术的飞速发展，研究人员可同时检测肿瘤组织中成千上万个基因的表达水平，从中发现与肿瘤组织起源相关的基因及特定的表达模式。转移灶肿瘤的基因表达谱与转移部位组织的基因表达谱存在差异，而与其原发部位组织的基因表达谱更相似，提示肿瘤在其发生、发展和转移的过程中，始终保留其组织起源的基因表达特征。根据这一原理，通过对转移灶的肿瘤组织中的基因表达水平进行检测，结合大数据的分析，可以精准的诊断转移灶的组织类型，为患者的精准治疗打下坚实的基础。

杭州可帮基因科技有限公司（Canhelp Genomics Co., Ltd）是国内专注于肿瘤 RNA 分子诊断技术的高科技企业，总部位于中国杭州。团队成员来自鲁汶大学、复旦大学、同济大学、生物梅里埃、诺华制药等顶尖研发机构和跨国公司，拥有核心研发能力与丰富行业经验。公司以基因表达谱检测和生物信息分析技术为核心，建立起行业领先的 RNA 分子诊断技术研发和转化平台，全面加速新一代肿瘤标志物的发现与产业化。公司研发管线聚焦肿瘤无创检查、辅助诊断、预后评估、用药指导等临床迫切需求，致力于开创癌症基因检测的新维度，为医生提供领先的诊断产品和服务，帮助患者实现癌症的精准治疗。

让医生有力量，让患者有希望，是我们一路前行的动力。

可帮全体员工

目录 CONTENTS

肿瘤组织起源检测报告

实验流程及相关信息

- 样本肿瘤细胞学质控
- 样本肿瘤细胞 RNA 基础信息
- 样本肿瘤细胞 RNA 扩增情况

多原发及不明原发肿瘤

肿瘤组织起源检测的临床价值

相关基因列表

局限性申明

参考文献

➢ 肿瘤组织起源检测报告

基本资料

患者姓名：刘艳华 年龄：41 性别：女

样本编号：GE90-23-DS-081458 病理编号：BI23-19750 送检时间：2023/11/27

检测结果

肿瘤组织起源检测结论：

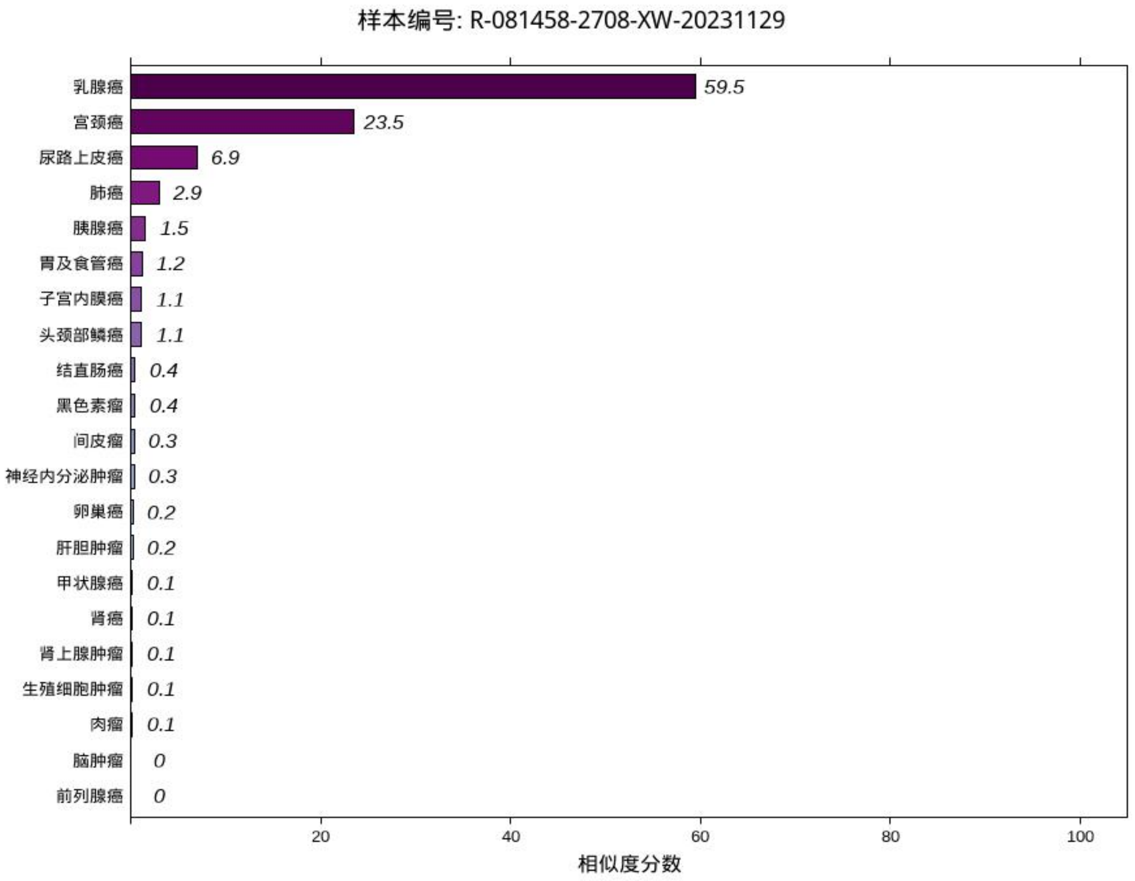

基因表达谱实验结果：乳腺癌

注：本检测结果不能作为肿瘤诊断的唯一依据，请结合病史、影像学、组织形态、免疫组化结果等临床病理信息综合判断检测结果的临床价值。

➢ 实验流程及相关信息

样本肿瘤细胞学质控

样本类型： ☑ 手术样本 ☐ 穿刺样本 活检部位： 右骨盆

样本种类： ☐ 新鲜组织 ☐ 冷冻组织 ☑ 石蜡切片 ☐ 蜡块 ☐ 蜡卷

送检组织量： 白片30张 肿瘤组织比例： 70%

既往肿瘤病史： 无

病理诊断： （右骨盆）浸润/转移性腺鳞癌，目前肿瘤细胞免疫表型无明确器官特异性，乳腺来源化生性癌、皮肤附属器来源腺鳞癌不能除外，请结合临床考虑肿瘤原发部位。

肿瘤形态学示意图：

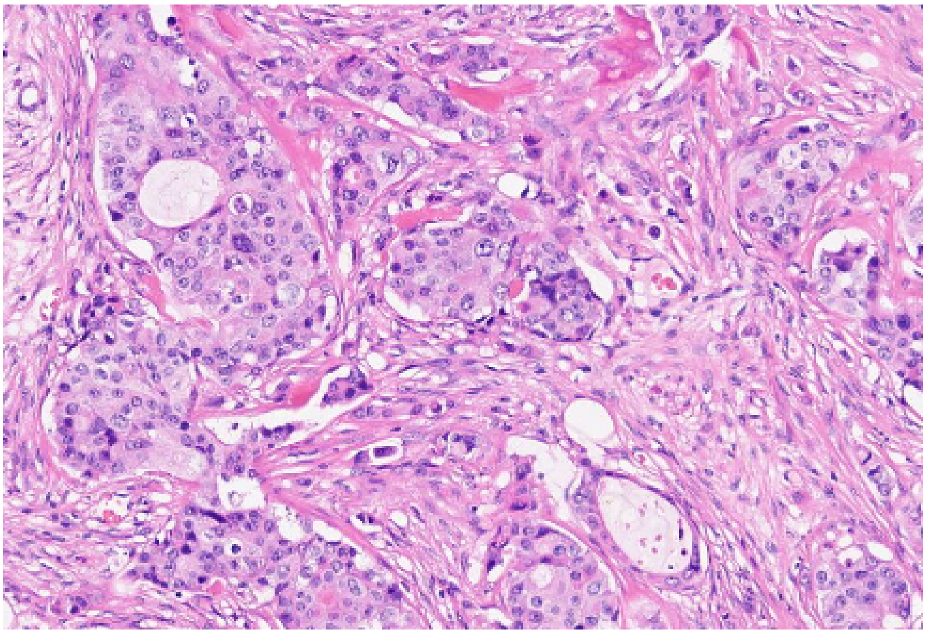

样本肿瘤细胞 RNA 基础信息

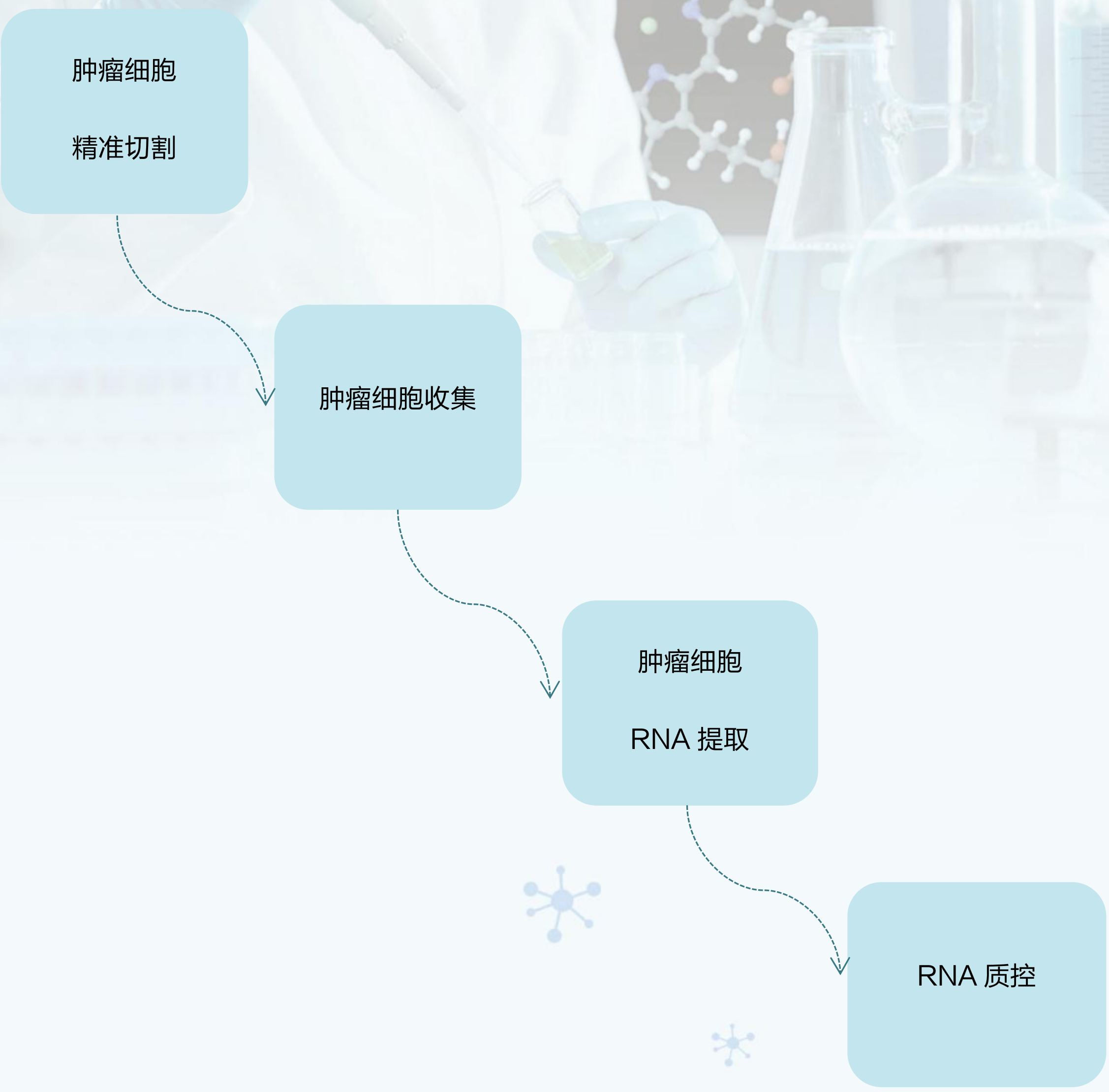

**肿瘤细胞 RNA 基础信息**	RNA 浓度	24.16 ng/μL
	RNA 纯度（A260/A280）	1.83
	内参 IC 值	26.89

样本肿瘤细胞 RNA 扩增情况

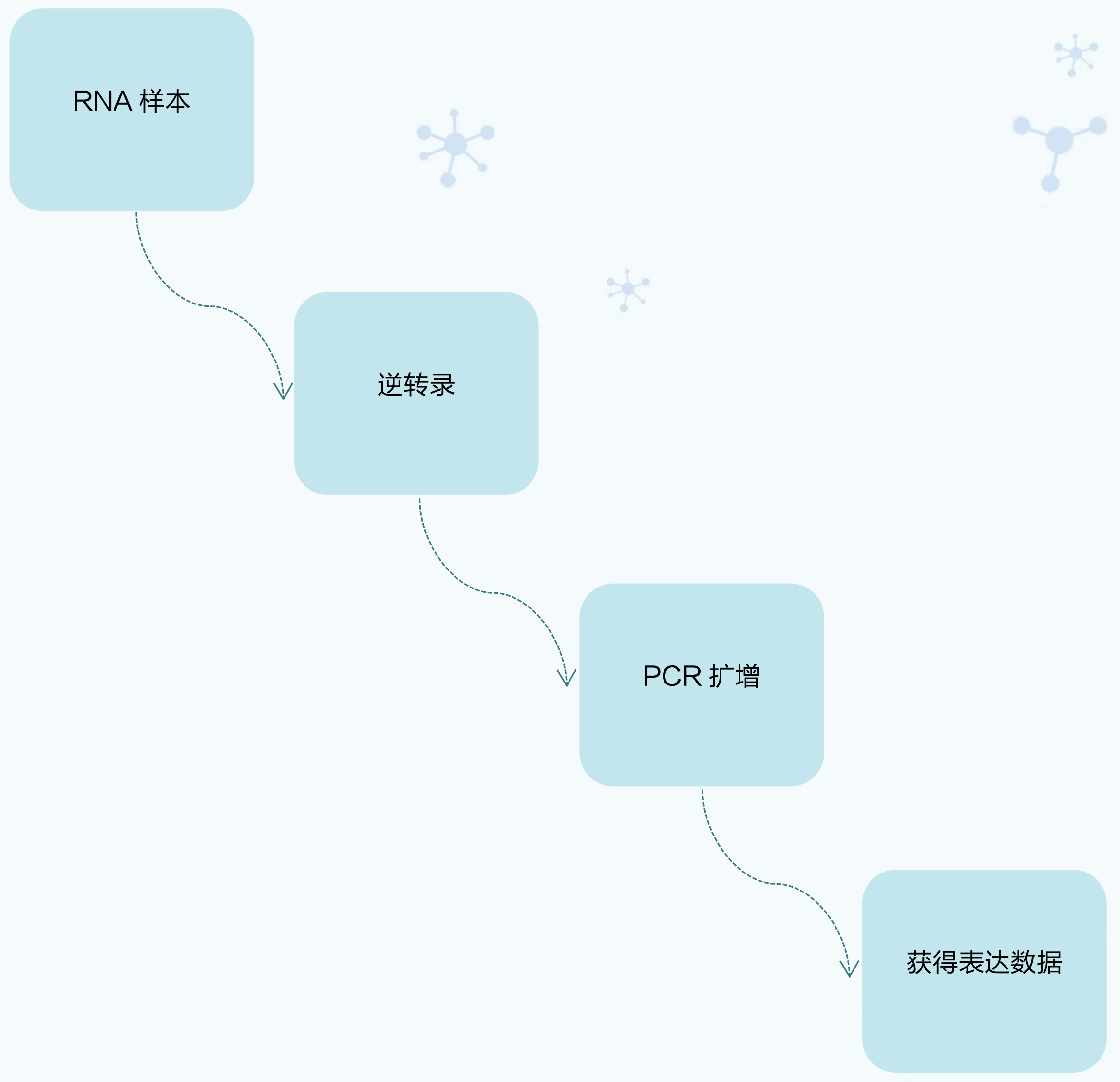

- **本样本的 90 基因扩增曲线图**

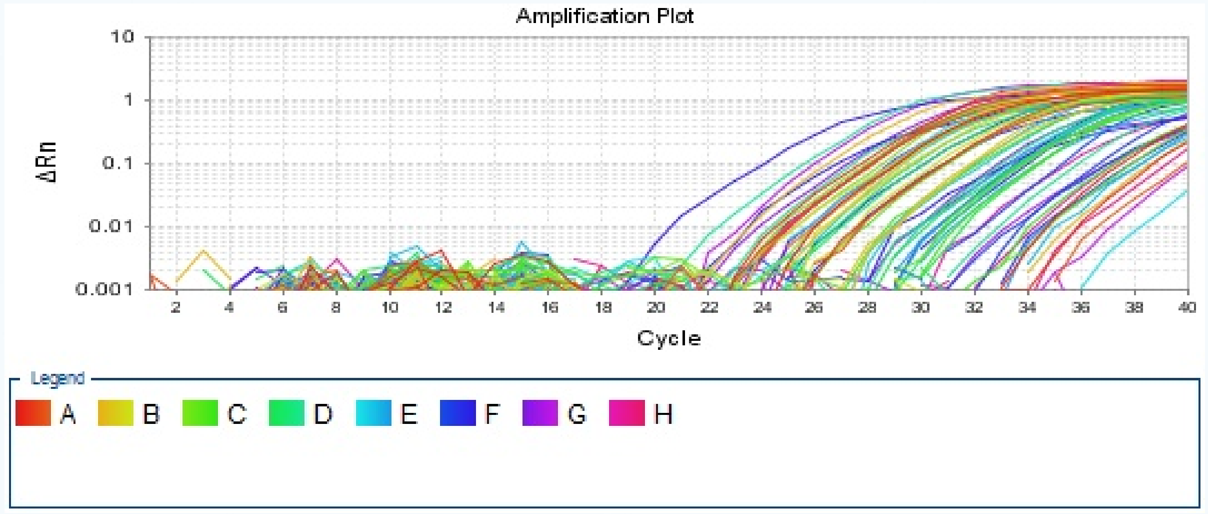

实验结论输出流程

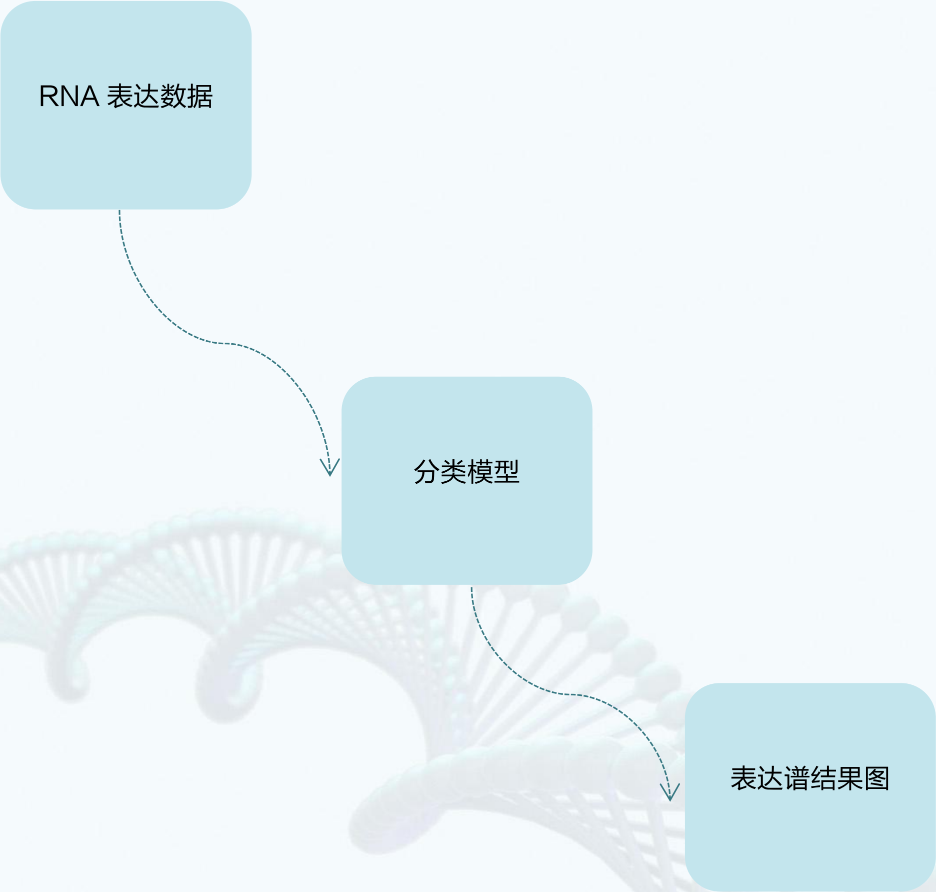

➢ 多原发及不明原发肿瘤

原发不明肿瘤(cancer of unknown primary，CUP)是一类经病理学诊断确诊为转移性恶性，但是通过详细评估未能明确原发位点的异质性肿瘤。原发不明肿瘤诊治困难，患者往往不知道去哪个科就诊，医生处理这类患者也非常棘手。近年来人们逐渐认识到原发不明肿瘤可能保留本身原发灶的生物学标记，而原发不明肿瘤的预后很大程度上取决于原发肿瘤的生物学特性，因此找出肿瘤的组织起源，采取有针对性的治疗，对于改善患者预后具有重要意义。

多原发肿瘤(cancer of multiple primaries，CMP)是指个体同时或先后发生两种或两种以上的原发性肿瘤。可以发生在同一器官或不同器官。由于以下一些原因，多原发肿瘤的发生率正在逐渐升高：诊断水平的提高；肿瘤治疗效果的改善，患者生存期的延长；放化疗引起的第二原发肿瘤等。然而，目前临床上对多原发肿瘤的认识存在很大不足，多原发肿瘤往往被误诊为转移性肿瘤，而其治疗效果往往好于转移性肿瘤。

临床实践中，CUP 和 CMP 的初步评估包括病史询问、完善的体格检查、实验室检查、内镜及影像学检查等。PET/CT 是目前最有效的影像学识别肿瘤原发位点的工具，诊断率在 24%～53%。病理组织学诊断，特别是免疫组织化学检查，是判断肿瘤组织起源的金标准。然而即使最好的专家运用最先进技术，只有 20%～30%的病例可明确原发灶，且病理诊断的主观性较强。

对肿瘤组织起源的精确诊断，一方面可以指导临床制定更有针对性的治疗方案，避免盲目的治疗选择，另一方面可以减少由于肿瘤类型不明确而给患者带来的巨大心理压力。

➢ 肿瘤组织起源检测的临床价值

近年来，随着分子生物学和基因组学的不断发展，科学家研究发现可以将几十个至几百个基因的表达水平作为分子指纹用于肿瘤的类型诊断，我们称之为肿瘤分子分型检测（MCCAs）。

随着芯片检测技术的发展，基于芯片平台的肿瘤分子分型检测开始运用于科研和临床领域，并且取得了不错的成绩：

1. 一项 149 例 CUP 患者的研究，免疫组化检测仅能对 35%的患者给出单一原发灶的判别，这其中有 77%患者的肿瘤分子分型检测结果与免疫组化结果相符合；在剩余 65%免疫组化无法给出明确结果的患者中，肿瘤分子分型检测仍然能够提示大部分患者的原发灶；

2. 经 MCCAs 检测判定为转移性结直肠癌的 CUP 患者（结肠镜检查为阴性）在接受结直肠癌针对性治疗后获得了超过 20 个月的中位生存期；

3. 一组 20 名通过 MCCAs 检测方法判定为肾细胞癌的 CUP 患者中（CT 影像无肾占位病变），在接受肾细胞癌针对性治疗后中位生存期为 16 个月。

杭州可帮基因科技有限公司联合复旦大学附属肿瘤医院共同开发了国内肿瘤分子分型检测技术—Canhelp® – Origin，该检测技术基于 qPCR 检测平台，通过检测 90 个肿瘤组织特异性表达的基因，对肿瘤的类型做出精确的判断。

研究人员对 Canhelp® – Origin 检测的有效性和可靠性进行了全面的测试。在包含 9578 例肿瘤样品的验证研究中，Canhelp® – Origin 对于 21 种肿瘤类型的平均准确度为 92.5%；其中在 11 种肿瘤中的灵敏度高于 90%，18 种肿瘤中灵敏度高于 80%，平均灵敏度为 91.1%；在 19 种肿瘤中的特异度高于 99%，平均特异度为 99.6%。

➤ 相关基因列表

基因缩写	基因名称	中文名称	相关肿瘤类型
ACPP	Acid phosphatase, prostate	酸性磷酸酶，前列腺	肝和前列腺
ACTG2	Actin, gamma 2, smooth muscle, enteric	肌动蛋白γ2	胃及食管, 间皮瘤, 和尿路上皮
AGR2	Anterior gradient 2, protein disulfide isomerase family member	蛋白质二硫键异构酶家族成员	卵巢
APOBEC3B	Apolipoprotein B mRNA editing enzyme, catalytic polypeptide−like 3B	载脂蛋白 B mRNA 编辑酶	肾上腺
APOD	Apolipoprotein D	载脂蛋白 D	胰腺
ASPN	Asporin	无孢蛋白	头颈
ATP1B1	ATPase, Na+/K+ transporting, beta 1 polypeptide	腺苷三磷酸酶	肾和尿路上皮
AZGP1	Alpha−2−glycoprotein 1, zinc−binding	α−2−糖蛋白 1，锌结合酶	乳腺
C7	Complement component 7	补体成分 7	卵巢
CA12	Carbonic Anhydrase 12	碳酸酐酶 12	肾
CDH1	Cadherin 1, type 1	钙粘蛋白 1	肉瘤
CDH17	Cadherin 17, LI cadherin (liver - intestine)	钙粘蛋白 17	结直肠
CEACAM5	Carcinoembryonic antigen−related cell adhesion molecule 5	癌胚抗原相关细胞粘附分子 5	乳腺，结直肠和子宫内膜
CEACAM6	Carcinoembryonic antigen−related cell adhesion molecule 6 (non−specific Cross −reacting antigen)	癌胚抗原相关细胞粘附分子 6	肺和尿路上皮
CHGA	Chromogranin A	嗜铬粒蛋白 A	神经内分泌
CHI3L1	Chitinase 3−like−1	几丁质酶 3	脑，肉瘤和尿路上皮
CLDN18	Claudin 18	紧密连接蛋白 18	胃及食管
CLU	Clusterin	丛生蛋白	脑

➢ 相关基因列表

基因缩写	基因名称	中文名称	相关肿瘤类型
COL11A1	Collagen, type XI, alpha−1	胶原蛋白 XI 型，α1	脑和子宫内膜
CXCL14	Chemokine (C−X−C motif) ligand 14	趋化因子受体 14	肝
CYP17A1	Cytochrome P450 family 17 subfamily A member 1	细胞色素 P450 家族 17 子族 A	肾上腺
DLK1	Delta−like 1 homolog (Drosophila)	δ 样同系物	神经内分泌和生殖细胞
EPCAM	Epithelial cell adhesion molecule	上皮细胞黏附分子	结直肠，肝，淋巴瘤和间皮瘤
ESR1	Estrogen receptor 1	上皮受体 1	子宫内膜
FABP1	Fatty acid−binding protein 1, liver	脂肪酸结合蛋白 1	结直肠
FABP4	Fatty acid−binding protein 4, adipocyte	脂肪酸结合蛋白 4	乳腺和肺
GATA3	GATA−binding protein 3	GATA 结合蛋白 3	乳腺和结直肠
GCG	Glucagon	胰高血糖素	胰腺
GFAP	Glial fibrillary acidic protein	胶质纤维酸性蛋白	脑
GJA1	Gap junction protein alpha−1	缝隙连接蛋白 α−1	宫颈
GPM6B	Glycoprotein M6B	糖蛋白 M6B	脑和黑色素瘤
GPX3	Glutathione peroxidase 3	谷胱甘肽过氧化物酶 3	甲状腺
GREM1	Gremlin 1, DAN family BMP antagonist	DAN 家族 BMP 拮抗剂	胃及食管
HBB	Hemoglobin subunit beta	血红蛋白亚基 β	脑和肉瘤
ID4	Inhibitor of DNA binding 4, dominant−negative helix −loop−helix protein	DNA 结合抑制剂 4	甲状腺
IGFBP2	Insulin−like growth factor binding protein 2	胰岛素生长因子结合蛋白 2	脑

相关基因列表

基因缩写	基因名称	中文名称	相关肿瘤类型
IGFBP7	Insulin–like growth factor binding protein 7	胰岛素生长因子结合蛋白 7	肾
IGJ	Joining chain of multimeric IgA and IgM	多聚体连结链	肺
ISL1	ISL LIM homeobox 1	ISL 同源序列 1	神经内分泌
KLK2	Kallikrein–related peptidase 2	激肽释放酶相关肽酶 2	前列腺
KLK3	Kallikrein–related peptidase 3	激肽释放酶相关肽酶 3	肝，前列腺和生殖细胞
KRT13	Keratin 13, type I	I 型角蛋白 13	头颈，黑色素瘤和尿路上皮
KRT14	Keratin 14, type I	I 型角蛋白 14	乳腺
KRT15	Keratin 15, type I	I 型角蛋白 15	头颈
KRT19	Keratin 19, type I	I 型角蛋白 19	肾上腺，头颈，淋巴瘤，间皮瘤和尿路上皮
KRT20	Keratin 20, type I	I 型角蛋白 20	结直肠
LGALS4	Lectin, galactoside–binding, soluble, 4	外源凝集素，结合型半乳糖苷 4	结直肠
LUM	Lumican	光蛋白聚糖	子宫内膜和卵巢
MGP	Matrix Gla protein	基质 Gla 蛋白	子宫内膜和卵巢
MMP1	Matrix metallopeptidase 1	基质金属蛋白酶 1	头颈
MMP12	Matrix metallopeptidase 12	基质金属蛋白酶 12	子宫内膜和卵巢
MMP3	Matrix metallopeptidase 3	基质金属蛋白酶 3	头颈
MSMB	Microseminoprotein–beta	微精元蛋白 β	前列腺
NKX3–1	NK3 homeobox 1	NK3 同源序列 1	前列腺

➢ 相关基因列表

基因缩写	基因名称	中文名称	相关肿瘤类型
NPTX2	Neuronal pentraxin II	神经穿透素 II	肾上腺
NPY1R	Neuropeptide Y receptor Y1	神经肽 Y 受体 Y1	肾
PCDH7	Protocadherin 7	原钙黏附蛋白 7	卵巢
PCP4	Purkinje cell protein 4	浦肯野细胞蛋白质 4	前列腺
PEG3	Paternally expressed 3	浦肯野细胞蛋白质 3	卵巢和生殖细胞
PI15	Peptidase inhibitor 15	肽酶抑制剂 15	淋巴瘤
PIGR	Polymeric immunoglobulin receptor	聚免疫球蛋白受体	胃及食管
PLA2G2A	Phospholipase A2 group IIA	磷脂酶 A2 组 IIA	肝和前列腺
POSTN	Periostin, osteoblast−specific factor	骨膜蛋白，成骨细胞特异因子	甲状腺
PRRX1	Paired−related homeobox 1	关联配对同源序列 1	子宫内膜
PTGDS	Prostaglandin D2 synthase	前列腺素 D2 合成酶	肝
PTN	Pleiotrophin	多效生长因子	脑和肉瘤
RPS11	Ribosomal protein S11	核糖体蛋白 S11	生殖细胞
RPS4Y1	Ribosomal protein S4, Y−linked 1	核糖体蛋白 S4，Y 串联 1	宫颈，头颈，肾，卵巢，前列腺和生殖细胞
S100A2	S100 calcium−binding protein A2	S100 钙结合蛋白 A2	尿路上皮
S100A8	S100 calcium−binding protein A8	S100 钙结合蛋白 A8	宫颈，淋巴瘤，间皮瘤和肉瘤
S100P	S100 calcium−binding protein P	S100 钙结合蛋白 P	尿路上皮
SCGB2A2	Secretoglobin, family 2A, member 2	分泌蛋白，家族 2A，成员 2	乳腺

➢ 相关基因列表

基因缩写	基因名称	中文名称	相关肿瘤类型
SERPINA3	Serpin peptidase inhibitor, clade A (alpha−1 antiproteinase, antitrypsin), member 3	丝氨酸肽酶抑制剂，分支 A，成员 3	脑，乳腺，肝和间皮瘤
SERPINB3	Serpin peptidase inhibitor, clade B (ovalbumin), member 3	丝氨酸肽酶抑制剂，分枝 B，成员 3	宫颈
SFN	Stratifin	人分层蛋白	肉瘤
SFRP1	Secreted frizzled−related protein 1	分泌性卷曲相关蛋白 1	宫颈
SFTPB	Surfactant protein B	表面活化蛋白 B	肺
SLC3A1	Solute carrier family 3 (amino acid transporter heavy chain), member 1	溶质运载蛋白家族成员 3	肾
SPINK1	Serine peptidase inhibitor, Kazal type 1	丝氨酸肽酶抑制剂，考佐尔 1 型	胃及食管和胰腺
SPP1	Secreted phosphoprotein 1	分泌型焦磷酸蛋白 1	肾和淋巴瘤
SST	Somatostatin	生长激素抑制剂	胰腺
SULT2A1	Sulfotransferase family 2A member 1	磺基转移酶家族 2A 成员 1	肾上腺
TACSTD2	Tumor−associated calcium signal transducer 2	肿瘤相关钙信号转导 2	结直肠，淋巴瘤和尿路上皮
TG	Thyroglobulin	甲状腺球蛋白	甲状腺
TH	Tyrosine hydroxylase	酪氨酸羟化酶	肾上腺和神经内分泌
TM4SF4	Transmembrane 4 L six family member 4	跨膜 4L 6 家族成员 4	肝和胰
TSPAN8	Tetraspanin 8	跨膜四蛋白	乳腺，结直肠和胃及食管
TYRP1	Tyrosinase−related protein 1	酪氨酸酶相关蛋白	黑色素瘤
VEGFA	Vascular endothelial growth factor A	血管内皮生长因子 A	肾
XIST	X−inactive−specific transcript (non−protein coding)	X−惰性−特异转录子（非蛋白编码）	宫颈，子宫内膜，胃及食管，头颈，卵巢和前列腺

局限性申明

1. 本次检测结果仅对该份送检样本负责；

2. 若对本次检测结果存在异议，请在 5 个工作日内与本公司联系；

3. 相关科学研究报道，肿瘤是一种动态变化的疾病，本次检测结果仅可作为一段时间内实施治疗的参考。若一段时间后患者疾病出现变化（包括疾病转好、稳定或进展等），建议患者再次咨询主治医生，科学制定治疗方案；

4. 本次检测结果仅供参考，最终治疗方案请遵医嘱。

参考文献

2018. Cancer of Unknown Primary Site: New Treatment Paradigms in the Era of Precision Medicine. John D. Hainsworth and F. Anthony Greco. Downloaded from ascopubs.org on July 17, 2018. American Society of Clinical Oncology.

2018. Renal Cell Carcinoma Presenting as Carcinoma of Unknown Primary Site: Recognition of a Treatable Patient Subset. Greco FA & Hainsworth JD, Clin Genitourin Cancer. Mar 9 epub ahead of print.

2017. 18F-FDG PET/CT for detection of the primary tumor in adults with extracervical metastases from cancer of unknown primary. A systematic review and meta-analysis. Burglin, Synne Alexandra et al. Medicine (2017) 96:16 Burglin 2017 F-FDG PET CT.

2017. Utility of Genomic Analysis In Circulating Tumor DNA from Patients with Carcinoma of Unknown Primary. Shumei Kato et al. American Association for Cancer Research. Published online First June 22, 2017. C Kato et al.

2017. Differences in experiences of care between patients diagnosed with metastatic cancer of known and unknown primaries: mixed-method findings from the 2013 cancer patient experience survey in England . Wagland, R; Bracher, M; Drosdowsky, A; Richardson, A; Symons, J; Mileshkin, L; Schofield, P. BMJ Open Volume 7, Issue 9.

2017. Ten years a CUP charity: is the unknown nearly known? Symons, John. CUP Foundation Paper. June 2017.

2017. Perceptions of Cancer of Unknown Primary site – A National Survey of Australian Medical Oncologists: Australian Medical Oncologist CUP survey. Christos S Karapetis et al. Internal Medicine Journal. Jan 2017.

2016. Lung Adenocarcinoma with Anaplastic Lymphoma Kinase (ALK) Rearrangement Presenting as Carcinoma of Unknown Primary Site: Recognition and Treatment Implications. Hainsworth JD, et al. Drugs Real World Outcomes.

2016. Improved diagnosis, therapy and outcomes for patients with CUP Nature Reviews, Clinical Oncology. Greco, F. Anthony. Nov. Kandalaft, PL & Gown, AM.

2016. Economopoulou P, Pentheroudakis G. Cancer of unknown primary: time to put the pieces of the puzzle together? Lancet Oncol. Oct;17(10):1339–1340.

2016. Practical applications in immunohistochemistry: carcinomas of unknown primary site. Kandalaft, PL & Gown, AM. Arch. Pathol. Lab. Med. 140, 508–523 (2016).

2017. Gene expression profiling identifies responsive patients with cancer of unknown primary treated with carboplatin, paclitaxel, and everolimus. Yoon, H. H. et al. Ann. Oncol. 27, 339–344 (2016).

2015. Paclitaxel/carboplatin with or without belinostat as empiric first-line treatment for patients with carcinoma of unknown primary site: A randomized, phase 2 trial. Hainsworth JD, et al. American Cancer Society.

2015. Fotopoulos G, et al. Prognostic significance of WNT and hedgehog pathway activation markers in cancer of unknown primary. Eur J Clin Invest. 2015 Nov;45(11):1145–52. Abstract.

2015. Economopoulou P, et al. Cancer of Unknown Primary origin in the genomic era: Elucidating the dark box of cancer. Cancer Treat Rev. May. Abstract.

2016. A case of S-100 negative melanoma: a diagnostic pitfall in the workup of a poorly differentiated metastatic tumor of unknown origin. Biernacka, A. et al. Cytojournal 13, 21 (2016).

2016. Pre-referral GP consultations in patients subsequently diagnosed with rarer cancers: a study of patient-reported data. Mendonca, Silvia C, Abel, Gary A, Lyratzopoulos, Georgios. British Journal of General Practice. 1 Mar 2016.

2016. Comparison of responses to closed questions from patients with a known primary versus unknown primary in the 2013 Cancer Patient Experience Survey (England). Schofield, P, Drosdowsky, A, Wagland, R, Symons, J, Mileshkin, L, Richardson, A, Ratchford, D, Bowtell, D, Gough, K. Australian Government. Cancer Australia.

2015. Experiences of Care of Patients with Cancer of Unknown Primary (CUP): Analysis of the 2010, 2011-12 & 2013 Cancer Patient Experience Survey. Wagland R, Bracher M, Ibanez Esqueda A, Schofield P, Richardson A. Southampton University, Health Sciences. Executive summary and Full report.

2015. Cancer of Unknown Primary During Pregnancy: an Exceptiona l ly Rare Coexistence. Nicholas Pavlidis, Fedro Peccatori, Fiona Lofts and Anthony F Greco. Anticancer Research 35: 575-580.

热线：400 880 9317
网址：www.canhelpdx.com
邮箱：info@cancerhelp.cn

可帮医学
Canhelp Medical

企业：杭州可帮医学检验实验室有限公司
地址：杭州市临平区新颜路 22 号浙江省生物医药孵化器
