# Supplementary figures and images for: Clinical application of 90-gene expression test in a patient with occult breast cancer: a case report and literature review
Source: Breast Cancer. 2025 Jun 5;32(5):1144–51. doi: 10.1007/s12282-025-01728-0 (PMC12394298; doi:10.1007/s12282-025-01728-0)

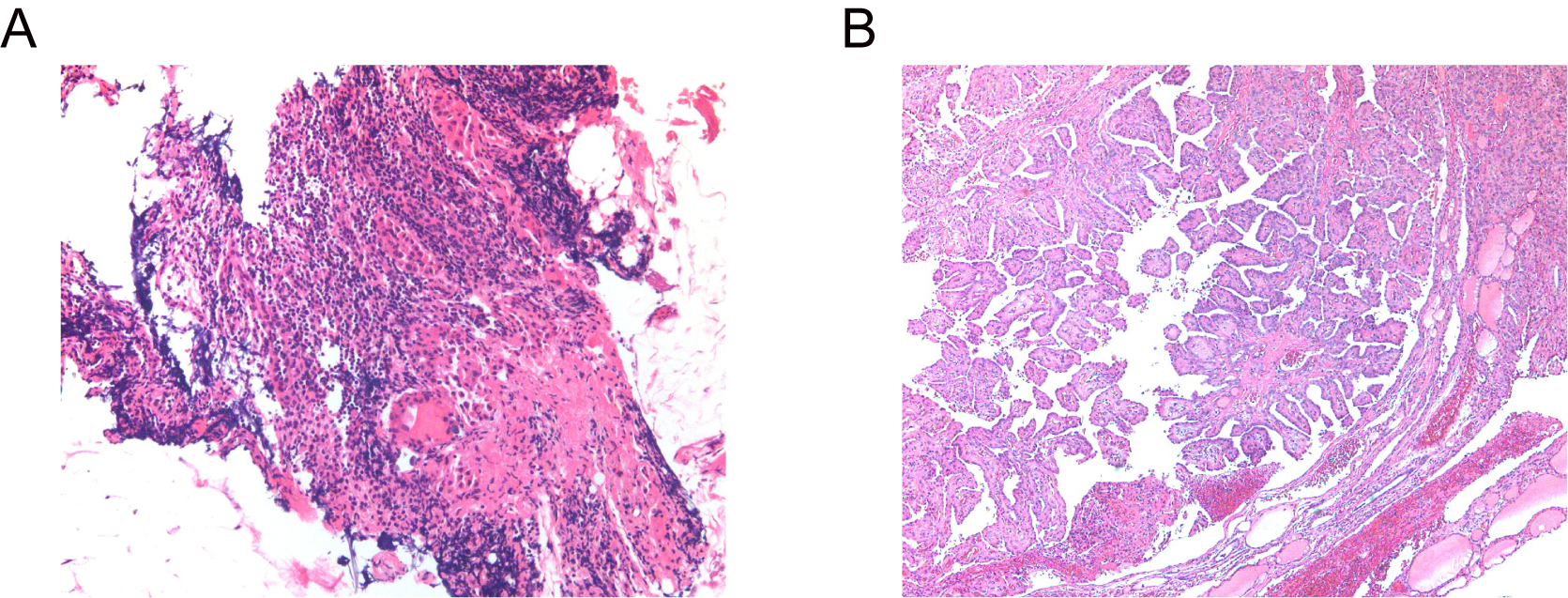

Supplement: Supplementary file 2 — Supplementary file2 (TIF 4540 KB) Supplementary Figure 1. The HE-stains results of thyroid surgery and diagnosis of papillary carcinoma of the thyroid. (A) HE*40; (B) HE*100 [file 12282_2025_1728_MOESM2_ESM.tif]
